# HTLV-1 bZIP factor: the key viral gene for pathogenesis

**DOI:** 10.1186/s12977-020-0511-0

**Published:** 2020-01-08

**Authors:** Masao Matsuoka, Jean-Michel Mesnard

**Affiliations:** 1 Department of Hematology, Rheumatology and Infectious Diseases, Faculty of Life Sciences, Kumamoto University, Kumamoto, 860-8556 Japan; 2 Laboratory of Virus Control, Institute for Frontier Life and Medical Sciences, Kyoto University, 53 Shogoin Kawahara-cho, Sakyo-ku, Kyoto, 606-8507 Japan; 3 IRIM, Université de Montpellier, CNRS, Montpellier, France

**Keywords:** HTLV-1, HBZ, Viral oncogenesis, Regulatory T cell

## Abstract

Human T cell leukemia virus type 1 (HTLV-1) causes adult T-cell leukemia-lymphoma (ATL) and inflammatory diseases. The HTLV-1 bZIP factor (HBZ) gene is constantly expressed in HTLV-1 infected cells and ATL cells. HBZ protein suppresses transcription of the *tax* gene through blocking the LTR recruitment of not only ATF/CREB factors but also CBP/p300. HBZ promotes transcription of Foxp3, CCR4, and T-cell immunoreceptor with Ig and ITIM domains (TIGIT). Thus, HBZ is critical for the immunophenotype of infected cells and ATL cells. HBZ also functions in its RNA form. HBZ RNA suppresses apoptosis and promotes proliferation of T cells. Since HBZ RNA is not recognized by cytotoxic T cells, HTLV-1 has a clever strategy for avoiding immune detection. HBZ plays central roles in maintaining infected T cells in vivo and determining their immunophenotype.

## Background

Complex retroviruses like Human T-cell leukemia virus type 1 (HTLV-1) harbor all three common retroviral genes (*gag*, *pol*, and *env*) in addition to both regulatory genes directly involved in regulation of viral expression and accessory genes. All these genes are expressed through transcripts initiating from the enhancer/promoter in 5′ Long Terminal Repeat (LTR) region. Furthermore, a crucial study published in 2002 demonstrated undeniably the existence of a negative-strand-encoded protein termed HBZ (HTLV-1 bZIP factor) [1] synthesized from antisense transcripts produced from the 3′ LTR [2–5]. Thereafter, it became evident that HBZ is the only viral gene conserved and expressed in adult T-cell leukemia-lymphoma (ATL) cells, indicating that HBZ plays critical roles in leukemogenesis [3]. In addition, HBZ is implicated in inflammatory diseases caused by HTLV-1 like HTLV-1-associated myelopathy/tropical spastic paraparesis (HAM/TSP). In this review, we summarize what is known about HBZ and discuss its significance in pathogenesis by HTLV-1.

## Transcriptional control of the *hbz* gene

Expression of the *hbz* gene is regulated by a bidirectional promoter located in the 3′ LTR [5–7]. It is noteworthy that all retroviral LTRs examined to date contain bidirectional promoters [7–12]. For HTLV-1, deletion mutants and point mutation experiments have revealed the importance of three Sp1-binding sites to be essential for antisense promoter regulation [5, 7]. Sp1 allows transcription to initiate from TATA-less promoters, and in fact HTLV-1 antisense transcripts initiate from multiple positions due to the absence of TATA boxes [3, 4].

A positive effect of Tax (HTLV-1 trans-activator) on antisense transcription has been suggested, based on data that were obtained from cells transfected with reporter vectors containing only one LTR [5, 7, 13]. On the other hand, results were totally different when analyzing sense and antisense transcription from viral constructs containing both LTRs: Tax did not activate antisense transcription from the 3′ LTR in this case [14]. Interestingly, transfection of a 5′ LTR-deleted proviral clone into 293T cells stimulates synthesis of antisense transcripts from the 3′ LTR [4], suggesting that decreased sense transcription may indeed result in an increased antisense transcription. This buildup of *hbz* mRNA in response to sense transcript decay has also been observed in short-term cultures of freshly isolated CD4^+^ T cells from HAM/TSP patients [15]. Conversely, analysis of viral protein expression in HTLV-1-infected T-cell lines confirmed very low HBZ levels in cells expressing elevated amounts of Gag [16, 17]. Taken together, all these observations suggest that Tax could indirectly keep antisense transcription to low levels through its stimulation of sense transcription and viral production. Studies performed using an in vivo rabbit model came to a similar conclusion. In HTLV-1-infected rabbits, entry into the chronic stage of infection coincides with the loss of Tax and Gag production, while HBZ expression is maintained at a steady state [16]. These results confirm an inverse correlation between sense and antisense transcription, and between Tax/Gag and HBZ expression, suggesting that HTLV-1 may use HBZ for the establishment of chronic infection [18].

## HBZ suppresses sense transcription

**Fig. 1:**
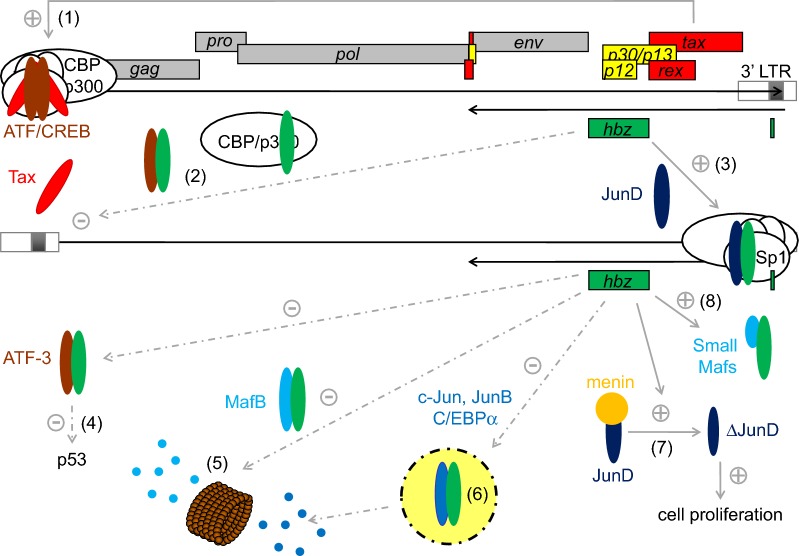
Effect of HBZ on bZIP factor activities. (1) Tax interacts with CREB to bind to vCREs and then recruits CBP (or p300) to activate viral transcription from the 5′ LTR. (2) HBZ inhibits Tax-dependent viral transcription by interacting with CREB and CBP/p300. (3) HBZ also stimulates its own expression by forming HBZ/JunD heterodimers capable of interacting with Sp1 bound to the 3′ LTR. (4) HBZ can also bind to ATF-3/p53 complexes, reducing ATF3′s ability to enhance p53 activity. (5 and 6) HBZ inhibits the MafB, C/EBPα, c-Jun, and JunB transcriptional activities by promoting their degradation via a proteasome-dependent pathway (5) or by sequestration into nuclear bodies (6). HBZ stimulates JunD activity (7) by inducing expression of ΔJunD, a JunD isoform that is unable to interact with the inhibitor menin. HBZ is also able to activate HMOX1 transcription by forming heterodimers with the small Mafs [42]

HBZ could be essential for maintaining latency in infected cells, likely by down-regulating viral expression from the 5′ LTR [1, 18–20]. The HTLV-1 LTR contains three viral cyclic AMP response elements (vCREs) allowing for transcription transactivation by Tax; the viral transactivator does not specifically bind to vCRE sites but instead interacts with cellular ATF/CREB factors like CREB and CREB-2 (Fig. 1) [21–26]. These transcription factors contain both a leucine zipper (ZIP) domain involved in protein dimerization and a basic region (immediately preceding the ZIP domain) required for their binding to the CRE site. After binding to the LTR vCREs, the Tax/CREB complex recruits the cellular coactivator CREB-binding protein (CBP) or its paralogue p300 to the viral promoter [21–26]. Once CBP or p300 is associated with the promoter, it bridges interactions between the Tax/CREB complex and the general transcription factor/RNA polymerase II complex. Additionally, both CBP and p300 possess a histone acetyltransferase (HAT) domain that plays an essential role in transcriptional activation by mediating acetylation of promoter-concealing nucleosomal histones.

Interestingly, a yeast two-hybrid screening of an HTLV-1-infected T-cell cDNA bank using the CREB-2 bZIP domain as bait successfully identified HBZ, which was shown to inhibit Tax-dependent viral transcription by interacting with CREB-2 and inhibiting the binding of CREB-2 to the vCREs [1]. An equivalent negative effect of HBZ has also been described for other ATF/CREB factors like CREB, CREM, and ATF-1 [19]. Thus, even if Tax expression were higher than HBZ expression in HTLV-1 infected cells, HBZ would block the formation of an active transcriptional complex on the viral promoter by specifically targeting these cellular factors. However, it has also been suggested that HBZ could be insufficient to block Tax activity in HTLV-1-infected cells expressing Tax strongly [27].

Interestingly, HBZ and Tax have opposite effects on many signaling pathways [28]. HBZ suppresses the NFκB, NFAT, and AP-1 pathways, whereas Tax can activate them. Conversely, HBZ activates the TGF-β/Smad pathway while Tax inhibits it. This reciprocal expression pattern could also fine-tune these signaling pathways, leading to the survival and proliferation of infected cells.

## HBZ structure and interaction with CBP/p300

Two isoforms of HBZ (206 and 209 residue-long proteins) have been characterized, but differences in their amino acid sequences are restricted to a small region at their N-termini and are not believed to cause major discrepancies in protein function [4, 5]. Indeed, the polypeptide domains involved in the transcription regulation function of HBZ are well conserved in both isoforms [8]. First, HBZ possesses an N-terminal activation domain containing two LxxLL-like motifs that interact directly with the KIX domain of CBP/p300 that is also recognized by Tax [20]. By interacting with these co-activators, HBZ interferes with Tax’s ability to interact with CBP/p300 and thus prevents their recruitment to the viral promoter (Fig. 1). Therefore, HBZ has a bipartite mechanism of repressing Tax-dependent HTLV-1 transcription activation: HBZ blocks the LTR recruitment of not only ATF/CREB factors but also CBP/p300. Moreover, HBZ interacts with other CBP/p300 domains, including both the HAT and C/H3 domains [29]. These interactions inhibit the HAT activity of p300/CBP, causing a reduction in p53 acetylation and a repression of p53 activity [30]. HBZ also contains three basic regions in its central portion that mediate its nuclear localization [31]. However, while HBZ was found in the nucleus in ATL cells [32], HBZ would localize exclusively in the cytoplasm of infected cells in asymptomatic carriers and HAM/TSP patients [33]. Finally, its C-terminal region encompasses a bZIP domain that plays a key function in regulating the activity of several bZIP factors. This domain is subdivided into a basic region involved in DNA-binding and a ZIP that forms coiled-coil interactions with similar domains found in other bZIP transcription factors like CREB and CREB-2 [1, 19], ATF-1, -2, and -3 [34, 35], C/EBPα and γ [35, 36], MafB [37], c-Jun and JunB [31, 38, 39]. The basic region of the HBZ bZIP domain diverges from corresponding regions in cellular bZIP factors [40], and consequently, heterodimerization between HBZ and these factors inhibits their association with cellular promoters [36–39, 41]. On the other hand, HBZ has recently been reported to activate transcription of the Heme Oxygenase 1 (HMOX1) gene by interacting with the small Mafs (MafF, MafG, or MafK) at Maf responsive elements located in an enhancer upstream of *HMOX1* [42]. Small Mafs differ from MafB in that they lack activation domain but they could form a complex capable of recruiting CBP/p300 by binding to HBZ [42].

## HBZ and the AP-1 family

Among these cellular bZIP-containing transcription factors, HBZ specifically targets members of the Jun (Jun, JunB and JunD) family; these factors, together with members of the Fos, ATF/CREB and Maf families, all belong to the activating protein-1 (AP-1) transcription factor family. The central role of the AP-1 family in cellular transcriptional regulation turns it into a epicenter of pathological signal relay in diseases, particularly in the context of leukemia and lymphoma [43, 44]. Several groups have reported that in fresh ATL cells, AP-1 was strongly deregulated [17, 45]. Among the Jun family members, JunD is highly expressed in ATL cells, while c-Jun expression is maintained at an undetectable level. Interestingly, HBZ is able to inhibit c-Jun activity (Fig. 1) both by sequestrating it into transcriptionally inactive nuclear bodies and by promoting its degradation via a proteasome-dependent pathway [39, 46]. Meanwhile, HBZ interacts with JunD to stimulate its own expression from the viral 3′ LTR (Fig. 1), as well as the transcription of cellular genes such as *hTERT* [6, 47]. By ChIP assays, it has been demonstrated that Sp1 transcription factors bound to the 3′ LTR or to the *hTERT* promoter act as docking sites for the heterodimer HBZ-JunD to stimulate gene expression.

JunD has been primarily thought of as a growth suppressor only because menin, a broadly expressed tumor suppressor, inhibits JunD transcriptional activity by interacting with its N-terminal domain [48]. In the absence of menin, JunD switches from a growth suppression to a growth promotion activity [49]. We have recently found that JunD could also promote proliferation and cell transformation when associated with HBZ [6, 17]. In addition, the intronless mRNA of *jund* can generate two protein isoforms using alternative translation initiation sites: full-length JunD (JunD-FL) and Δ-JunD, an N-terminal truncated form of JunD-FL, unable to interact with menin. HBZ is able to stimulate Δ-JunD translation by depleting the ribosomal protein S25 [17] and thus can potentially mediate a shift from the JunD-FL-induced suppression of cell proliferation to promoting proliferation by stimulating Δ-JunD synthesis (Fig. 1).

## HTLV-1 infected cells and ATL cells maintain the *HBZ* gene

The HTLV-1 provirus is the only direct evidence of infection in ATL cells. Therefore, the analysis of HTLV-1 provirus in ATL cells provides us important information on leukemogenesis [50]. Transcription of *tax* is frequently undetectable in ATL cases. There are three mechanisms that inactivate Tax expression: (1) deletion of the 5′LTR [51, 52], (2) DNA methylation of the 5′LTR [53, 54], and 3) genetic changes (non-sense mutations, deletions or insertions) within the *tax* gene [55, 56]. Tax is not expressed in approximately half of ATL cases [57]. However, the pX region and the 3′LTR, which together contain the promoter and the coding region of the *HBZ* gene, remain intact in all ATL cases. Indeed, the *HBZ* gene is transcribed in all ATL cases [3]. In addition, the knock-down of HBZ suppresses the proliferation of ATL cells, indicating that the *HBZ* gene is critical for leukemogenesis [3, 58, 59].

When during the course of ATL leukemogenesis is Tax expression inactivated? Among genetic changes of the *tax* gene in ATL cells, non-sense mutations are the most frequently detected. Importantly, most non-sense mutations are found in TGG (methionine) codons, which are also the target sequences of APOBEC3G [56]. APOBEC3G targets single stranded DNA during reverse transcription and generates non-sense mutations in the provirus. Indeed, non-sense mutations of the *tax* gene are also found in the provirus of asymptomatic carriers, which indicates that these non-sense mutations are generated before proviral integration. Since most infected cells have only one copy of the provirus [60], ATL cells with non-sense mutations in the *tax* gene must have become transformed without Tax.

A similar important finding is that in some ATL cases, the 5′LTR is lost before integration. HTLV-1 integrase generates 6 bp short repeats at the 5′ and 3′ ends of the provirus. In some ATL cases without the 5′LTR, this short repeat is connected to an internal sequence (*pol* and *env*) of the provirus, and the 3′LTR, suggesting that viral integrase sometimes recognizes an internal sequence and the 3′LTR, and then integrates a defective provirus into the host genome [52]. Again, this shows that Tax expression can be lost before integration. The 3′LTR and HBZ coding region remain intact even in these defective proviruses in ATL cases, suggesting that in these cases, ATL may develop in the presence of HBZ alone.

## How HBZ modulates infected cells

The receptor for HTLV-1 is glucose transporter 1 (GLUT-1), neuropilin and heperan sulfate proteoglycan, which are expressed on a variety of cells. Indeed, HTLV-1 can infect many different types of cells, suggesting that infection itself does not define cell specificity. However, HTLV-1 provirus is detected mainly in CD4 + T cells in vivo [61]. Furthermore, the immunophenotype of most HTLV-1 infected cells is CD4 + CD45RO + CD25 + CCR4 + CADM1 + . Foxp3 is frequently expressed in HTLV-1 infected cells and ATL cells [62]. Thus, this virus targets a limited subpopulation of CD4 + T cells in vivo. Since this observation is not consistent with the findings that HTLV-1 can infect many different types of cells in vitro, it appears that HTLV-1 modulates the immunophenotypes of infected cells and/or promotes the proliferation of specific kinds of CD4 + T cells.

**Fig. 2:**
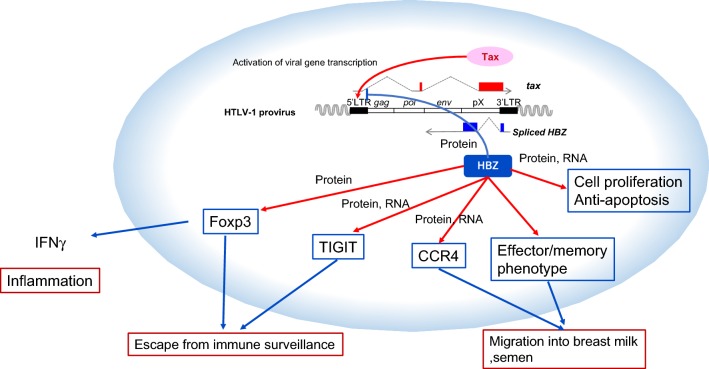
Functions of HBZ. HBZ induces transcription of Foxp3, CCR4 and TIGIT genes. HBZ expression promotes the proliferation of T cells and inhibits their apoptosis. In addition, HBZ changes expressing T cells to an effector/memory phenotype, which is important for their migration into breast milk and semen

It has been reported that HTLV-1 infects hematopoietic stem cells in the bone marrow, since identical integration sites of HTLV-1 provirus were identified in different hematopoietic cells [63]. One speculates that infected cells differentiate into various hematopoietic cells including T cells, B cells, monocytes, and neutrophils. HTLV-1 infection to immature cells was also suggested by the report that ATL clones with the identical integration site of the provirus and different T-cell receptor gene rearrangement were found in the same patients [64]. It is possible that viral gene(s) modulate the differentiation of infected cells. Since only HBZ is constantly expressed in infected cells, it is thought that HBZ must modulate this differentiation, increasing the population of infected CD4 T cells with specific markers. As mechanisms, HBZ induces transcription of Foxp3, CCR4, and T-cell immunoreceptor with Ig and ITIM domains (TIGIT) in vitro and in vivo (Fig. 2).

Why does HBZ convert infected cells to cells with such specific immunophenotypes? One possibility is that effector/memory T cells tend to enter breast milk and semen [28]. Since HTLV-1 transmits mainly through breast feeding and sexual intercourse, producing a large number of infected cells with this immunophenotype is critical for the transmission of HTLV-1 [65]. Another reason is to evade host immune responses. Foxp3 can induce expression of immunosuppressive molecules, including CTLA-4, GITR, IL-35 and IL-10. Presumably, HTLV-1 infected T cells that are Foxp3 + would tend to escape from host immune surveillance. Indeed, HTLV-1 provirus is detected in Foxp3 + CD4 + T cells in carriers.

## Function of *HBZ* RNA

Not only HBZ protein, but also *HBZ* RNA promotes the proliferation of infected cells. An *HBZ* mutant (TTG mutant), in which the first ATG is replaced by TTG, cannot produce the HBZ protein [3]. Interestingly, this TTG mutant still promotes the proliferation and inhibits the apoptosis of expressing T cells, implicating *HBZ* RNA in cell proliferation and ATL [66]. This function of *HBZ* RNA is particularly beneficial for HTLV-1 infected cells and ATL cells, because RNA is not recognized by the host immune surveillance. In the case of Epstein–Barr virus (EBV), it has been reported that EBV encoded small RNA (EBER) contributes to oncogenesis by modulating innate immunity [67]. Thus, functional viral RNA is thought to be clever strategy by which chronic viral infections evade host immune surveillance.

Tax is intermittently expressed in ATL cell lines and HTLV-1 infected cell lines [68]. This intermittent expression is associated with resistance to apoptosis. HBZ is also reported to be expressed in a part of HTLV-1 infected cells in contrast to ATL cell lines [69], in which *HBZ* is expressed in almost all cells. Level of *HBZ* transcripts, but not *tax*, is correlated with the S and G2/M phases of the cell cycle [69], suggesting that HBZ RNA, but not tax, is linked with cell proliferation [66].

## Implication of HBZ in leukemogenesis

In vivo expression of *HBZ* in transgenic mice causes T-cell lymphomas and inflammatory diseases [70]. Interestingly, *tax* transgenic mice in which *tax* is expressed in CD4 + T cells did not develop any such diseases [71]. Furthermore, Tax is not expressed in approximately half of ATL cases due to genetic changes of the *tax* gene, DNA methylation of 5′LTR and deletion of 5′LTR [50]. Importantly, non-sense mutations of the tax gene, and half of 5′LTR deletion are generated at infection of HTLV-1, suggesting that tax gene is not necessary for leukemogenesis in these cases [52, 56]. These findings suggest that HBZ, but not tax, is mainly implicated in pathogenesis by HTLV-1. Tax might play an important role in half of ATL cases since they retain capability to express Tax, and transient Tax expression is critical for survival of ATL cells [68]. In addition, HBZ induces genetic instability in expressing cells [72, 73], suggesting that HBZ promotes leukemogenesis by induced genetic instability.

A recent study using screening by gene knockout with the CRISPR/Cas9 system reported that IRF4 and BATF3 are critical for the growth of ATL cells [59]. HBZ protein upregulates the expression of BATF3. Mutation of IRF4 is also reported to be associated with poor prognosis for ATL patients [74]. Thus, HBZ appears to be responsible for leukemogenesis of ATL.

Comprehensive studies of genetic and epigenetic changes in ATL cells revealed that multiple alterations are identified in genes associated with T-cell receptor-NFκB signaling, T-cell tracking, other T-cell related pathways and immunosurveillance [75]. These pathways are also influenced by HBZ. In the carrier state, only the *HBZ* gene is constantly expressed, and ATL develops in the absence of Tax. But most infected cells do not become leukemic. Therefore, it is thought that genetic and epigenetic changes must potentiate or fix the effects of HBZ, leading to the development of ATL.

*HBZ* RNA is implicated in proliferation of ATL cells and HTLV-1 infected cells [3, 66]. Effects of HBZ protein and mRNA differ in cellular signaling pathways. *HBZ* RNA promotes cell proliferation and suppresses apoptosis whereas HBZ protein increases apoptosis [66]. However, both *HBZ* RNA and the protein induces expression of CCR4 and TIGIT. Thus, detailed function of *HBZ* RNA and the protein should be studied in the future.

## Future direction of HBZ research

It is becoming clear that HBZ plays several central roles in pathogenesis by HTLV-1. It is assumed that the pleiotropic functions of HBZ reflect a viral strategy that optimizes cell-to-cell transmission. The immunophenotypes of effector/memory T cells and Tregs are manipulated to allow the migration of infected cells into breast milk and semen—a phenomenon that also confers an infiltrative phenotype to ATL cells. However, questions remain about how *HBZ* RNA functions and how HBZ transforms T cells. In addition, there is the hope that immunotherapy against HBZ might improve the prognosis of ATL patients.

## Data Availability

Not applicable.

## Abbreviations

HTLV-1: human T-cell leukemia virus type 1
ATL: adult T-cell leukemia-lymphoma
HBZ: HTLV-1 bZIP factor
TIGIT: T-cell immunoreceptor with Ig and ITIM domains
LTR: long terminal repeat
HAM/TSP: HTLV-1-associated myelopathy/tropical spastic paraparesis
vCREs: viral cyclic AMP response elements
CBP: CREB-binding protein
HAT: histone acetyltransferase
GLUT-1: glucose transporter 1
EBV: Epstein–Barr virus
EBER: EBV encoded small RNA
